# Migraine: Calcium Channels and Glia

**DOI:** 10.3390/ijms22052688

**Published:** 2021-03-07

**Authors:** Marta Kowalska, Michał Prendecki, Thomas Piekut, Wojciech Kozubski, Jolanta Dorszewska

**Affiliations:** 1 Laboratory of Neurobiology, Department of Neurology, Poznan University of Medical Sciences, 49 Przybyszewskiego St., 60-355 Poznan, Poland; martak_89@o2.pl (M.K.); mprendecki@ump.edu.pl (M.P.); 82820@student.ump.edu.pl (T.P.); 2 Chair and Department of Neurology, Poznan University of Medical Sciences, 49 Przybyszewskiego St., 60-355 Poznan, Poland; wkozubski@ump.edu.pl

**Keywords:** migraine, FHM, cortical spreading depression, *CACNA1A*, CaV2.1, glia

## Abstract

Migraine is a common neurological disease that affects about 11% of the adult population. The disease is divided into two main clinical subtypes: migraine with aura and migraine without aura. According to the neurovascular theory of migraine, the activation of the trigeminovascular system (TGVS) and the release of numerous neuropeptides, including calcitonin gene-related peptide (CGRP) are involved in headache pathogenesis. TGVS can be activated by cortical spreading depression (CSD), a phenomenon responsible for the aura. The mechanism of CSD, stemming in part from aberrant interactions between neurons and glia have been studied in models of familial hemiplegic migraine (FHM), a rare monogenic form of migraine with aura. The present review focuses on those interactions, especially as seen in FHM type 1, a variant of the disease caused by a mutation in *CACNA1A*, which encodes the α1A subunit of the P/Q-type voltage-gated calcium channel.

## 1. Review Criteria

Articles discussed in this Review were identified by PubMed searches for the years 1990 to the present, using the search terms “migraine and calcium”, “migraine and calcium signaling”, “CACNA1A mutations”, “migraine and CaV2.1”, “migraine and glia”, “glia and calcium in migraine” among others. The reference lists of identified papers were searched for further relevant articles, and related citations for identified papers as listed on the PubMed site were also evaluated.

## 2. Migraine

Migraine is a common primary headache disorder that affects 11% of adults worldwide. The prevalence of disease is three times higher in women (15–18%) than in men [1]. Two peaks of incidence have been observed among migraine sufferers: the first after puberty and the second in adults aged 35–40 years [2]. In 25% of cases, the migraine begins in childhood. According to World Health Organization (WHO) data 324 million people struggle with this disease and 3000 migraine attacks occur every day per one million people [3]. The Global Burden of Disease study listed migraine as the third of 289 of the most prevalent diseases worldwide [4].

The disease is divided into two main clinical subtypes: migraine with aura (MA) and migraine without aura (MO). Aura is defined as spreading neurological disturbances such as visual, sensory or motor symptoms that precede or accompany the headache. The most common clinical features of aura are visual changes including flashing scotoma, loss of vision, and visual hallucinations. More rarely, numbness, tingling, ataxia, aphasia, confusion, ringing in the ears, and dizziness occur. MO, also called *common* migraine, occurs in two-thirds of patients. The migraine pain lasts 4–72 h, is moderate or severe, unilateral, throbbing, worsens with physical activity, and is often accompanied by photophobia, phonophobia, and nausea/vomiting [5,6,7,8]. Migraine is also classified according to the frequency of attacks into chronic, lasting at least 15 days each month, and milder, episodic forms. Chronic migraine, sometimes called transformed, affects about 2% of the population and occurs after many years of a typical, episodic migraine. The tendency to transformation may be increased by coexisting depression, anxiety, panic attacks, social phobia and by other pain syndromes [4,9].

### 2.1. The Pathogenesis of Migraine

**Figure 1:**
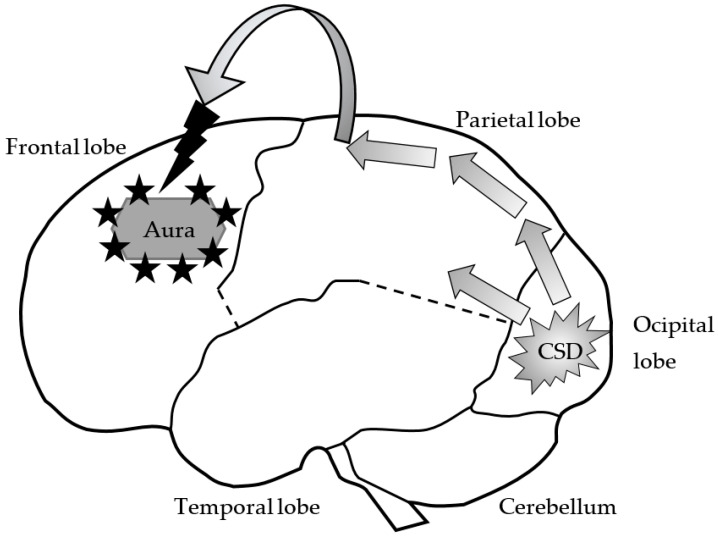
Cortical spreading depression. Changes in central nervous system flow that may induce a migraine attack may be associated with the occurrence of spreading cortical spreading depression (CSD). Oligemia (reduced vascular flow) begins in the occipital and parieto-occipital areas, then moves through the cerebral cortex and stops at the medial and lateral sulcus. In some patients, CSD may extend to the frontal lobes. This phenomenon seems to be responsible for the aura formation during a migraine headache.

Migraine pathophysiology involves complex mechanisms in which the trigeminovascular system (TGVS) and cortical spreading depression (CSD) play an important role (Figure 1) [10]. The TGVS is a major afferent pathway for pain from cranial vessels and dura mater and consists of neurons whose bodies reside in the trigeminal ganglion (TG) and upper cervical dorsal root ganglia [11]. The TG consists mainly of primary afferent neurons and glial cells. As it is not protected by the blood–brain barrier (BBB), neuropeptides released in the TG, such as calcitonin gene-related peptide (CGRP), substance P (SP), and neurokinin A (NKA) thereby enter systemic circulation [12].

CSD consists of a slow wave of depolarization followed by longer-lasting suppression of neurons and glial cells. It is characterized by increased K^+^ and decreased Na^+^ extracellular levels and changes in the gradients of other ions, e.g., Mg^2+^, Zn^2+^, Cl^−^ [13]. Although the direct triggers for spontaneous CSD, especially in migraine, are unknown, elevated extracellular concentrations of K^+^ and glutamate are thought to cause dendritic depolarization in a non-synaptic manner. Animal model studies have suggested that CSD is the mechanism underlying visual aura in migraine [14], a conclusion corroborated by neuroimaging studies which have linked electrical changes in the visual cortex during aura (geometrical shapes, scintillating scotoma) with the pattern of CSD [15].

According to the neurovascular theory of migraine, headache is a result of TGVS activation by CSD. CSD triggers the pain pathway via activation of trigeminal afferents which transmit information to the TG, the caudal trigeminal nucleus (TNC) and ultimately to cortical and brainstem structures involved in pain processing (Figure 1). Activation of the TGVS leads to local release of vasodilators such as CGRP, SP, NKA, NO, and a transient increase in cortical blood flow followed by sustained flow decrease [16,17,18].

### 2.2. Migraine Disturbs Calcium Homeostasis

Currently, it is believed that the distribution of various ions between intracellular and extracellular compartments is altered in migraine. As aforementioned, increased extracellular K^+^ and decreased Na^+^ promote CSD [13]. Calcium levels are also altered in the course of migraine [19]. These findings have led scientists to argue that migraine is a channelopathy. It is believed that mutations in genes encoding channel subunits or proteins modulating channel function lead to ionic disturbances in synapses, thereby increasing susceptibility to CSD and migraines [20,21,22].

**Figure 2:**
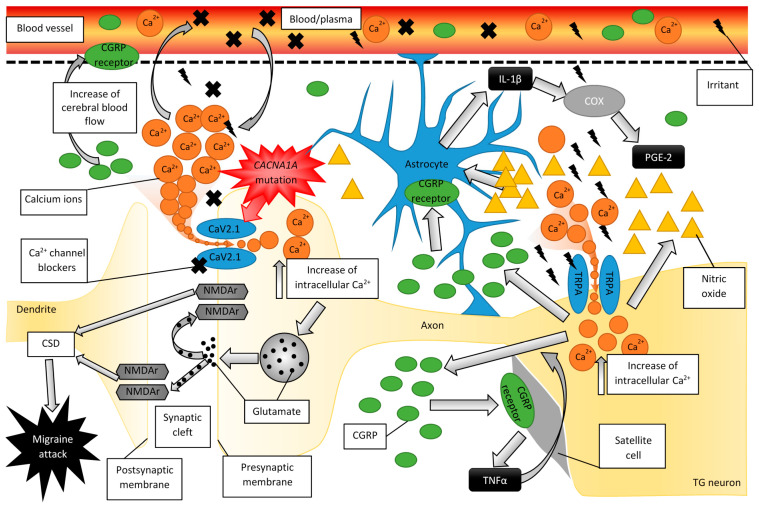
Induction of migraine attack due to sensitization of trigeminal ganglia and disturbed glutamatergic release. TRPA1 receptors in sensory neurons in trigeminal ganglia (TG) activate under environmental irritants taken by inhalation, ingestion or in an unknown mechanism. Activation of the TRPA1 receptor stimulates the release of calcitonin gene-related peptide (CGRP) that may enter bloodstream during migraine attack. CGRP receptor is expressed by majority on the neurons forming myelinated TG A-fibers, as well as on smooth muscles of dura’s vasculature. CGRP receptor stimulation increases blood flow. Besides CGRP, activated TG neurons secrete nitric oxide (NO). These mediators stimulate further the surrounding glial cells to produce interleukin-1β (IL-1β) that in turn leads to increased activity of cyclooxygenase (COX), associated with production of proinflammatory prostaglandin E2 (PGE2). This phenomenon may be one of foundations of the TG neurons sensitization. Subsequently, CGRP released by TG neurons may also promote release of tumor necrosis factor-α (TNF-α) in the glial satellite cells. That may cause a positive feedback loop of further TG-neuronal synthesis and secretion of CGRP. Furthermore, the released TNF-α may itself sensitize TG neurons and inflict overproduction of various other proinflammatory cytokines. Activation of TRPA channels increase the intracellular calcium ion levels (Ca^2+^), similarly to L-type of Voltage Gated Calcium Channel (CaV2.1). Mutations in *CACNA1A* gene lead to enhanced Ca^2+^ currents. The enhanced Ca^2+^ influx lead to excitation-inhibition imbalance and enhanced glutamate release. The interaction between glutamate and pre- and postsynaptic glutamate NMDA receptors (NMDAr) may facilitate the cortical spreading depression (CSD)—believed as one of the causes of migraine attack. Ca^2+^ channel blockers affect the CaV2.1 channels and reduce excessive Ca^2+^ influx to the cells, normalizing glutamate release and thus may be also used in the treatment of migraine headaches.

Dysregulated calcium currents as seen in the context of migraine derive largely from aberrant function of the high-voltage activated calcium channel CaV2.1 and the transient receptor potential ankyrin channel, encoded by the genes *CACNA1A* and *TRPA1*, respectively (Figure 2) [20,21,23]. Localized presynaptically, the CaV2.1 channel plays an important role in communication between neurons by controlling the release of neurotransmitters [24]. Certain mutations in the *CACNA1A* gene result in increased activation of CaV2.1, which in turn leads to increased intracellular Ca^2+^ [20,21,22,25]. TRPA1, a nonselective Ca^2+^-permeable ion channel, belongs to a family of transient potential receptors serving as modalities for the sensation of environmental stimuli. Expressed on Aδ and C afferent fibers, TRPA1 transduces pain from a broad array of irritants, both food (e.g., allyl isothiocyanate in mustard) and chemical (e.g., formaldehyde) and is thought to be implicated in the pathogenesis of headache [23,26]. Although the mechanisms whereby environmental irritants cause headache remain largely unknown, activation of TRPA1 by mechanical or chemical stimuli causes CGRP to be released and increases cerebral blood flow [26,27].

Beyond CaV2.1, other voltage-gated calcium channels (VGCCs) may play a role in the pathogenesis of migraine [28]. Interestingly, while presynaptic CaV2 channels might be expected to drive the release of CGRP associated with migraine, the high-voltage activated and canonically postsynaptic CaV1 channels and the low-voltage activated CaV3 channels [24,29] were both also found to regulate CGRP release in the trigeminal ganglion, as evidenced through pharmacological blockade experiments [30]. Further, by correlating the genetic codependency of Ca^2+^ levels with the risk of migraine headache, Yin et al. [31] showed that migraine can be linked to inherited hypercalcemia.

Several classes of drugs are currently used in the treatment and prevention of migraine, including angiotensin converting enzyme inhibitors, angiotensin receptor blockers, Ca^2+^ channel blockers, serotonin antagonists, alpha adrenergic agonists, and NMDA receptor antagonists [32]. Ca^2+^ channel blockers exert their effect by blocking Ca^2+^ influx into vascular smooth muscle and cardiac muscle cells during membrane depolarization, leading to a reduction in blood pressure. Their main application is in the treatment of arterial hypertension and angina and while used also for migraine pain relief therapy, they may themselves result in side effects such as headaches and dizziness. As Ca^2+^ channel blockers are specific for different VGCCs and vary broadly in their pharmacologic effects, the development of therapeutics that selectively target those channels which are centrally expressed and implicated in migraine pathogenesis is of high research value.

### 2.3. Experimental Evidence for the Role of CGRP in Migraine

Literature reports suggest that CGRP may be a principal mediator of migraine in the TGVS [33]. The CGRP neuropeptide is expressed in nearly half of TG neurons, mostly in those forming unmyelinated nociceptive C-fibers. Conversely, the CGRP receptor is expressed by the majority of neurons forming the second class of TG fibers, myelinated A-fibers, as well as in smooth muscle tissue of the dura’s vasculature [34]. There are two isoforms of CGRP, differing by three amino acids and the tissues where they are predominantly expressed. α-CGRP is most abundant in the CNS and peripheral nerves of the somatosensory system, while the β-isoform is mostly present in the enteric nervous system and motor neurons [35]. The connection between CGRP and migraine has been documented quite well, however a clear mechanistic explanation for its role in the disease is lacking. It has been shown that CGRP administered intravenously induces a delay in the onset of a migraine attack in individuals with a history of migraine, but not in healthy controls. Notably, CGRP is not allogenic, since it does not provoke any somatic effects beyond headache [36] or an erythemic flare in the skin [37]. Interestingly, while CGRP infusion was shown to induce delayed headache in patients suffering from migraines, the neuromodulator did not cause premonitory symptoms, such as aura, suggesting attacks may be periphery-derived [38]. Moreover, CGRP is likely released into the bloodstream during a migraine attack, especially seeing how it has been detected in samples drawn from the jugular vein of sufferers [16,36,37,38,39,40,41]. Increased CGRP levels have also been observed between attacks in migraine patients as compared to controls. As such, CGRP may be associated with the chronic form of migraine, particularly given that in this disease subtype, the level of the neuropeptide was significantly higher than in episodic migraine patients [41]. The level of CGRP was also shown to be elevated in a nitroglycerin model of migraine, which responded to the triptan family of drugs commonly used as abortive therapy for the disease [42]. Specifically, administration of sumatriptan significantly reduced CGRP release from the TG, and secondarily caused significant decline of blood CGRP levels, along with reduced headache intensity. Another study evaluating rizatriptan correlated higher salivary CGRP levels with better response to the drug, thereby potentially providing a way for identifying promising patient candidates for specific anti-migraine therapies [43]. Taken together, the above studies indicate a pivotal role of CGRP in TGVS regulation of migraine attacks and indeed, CGRP is becoming a target point for emerging therapies. Several clinical studies tackling the CGRP pathway for relieving migraine showed promising results and involve CGRP antagonists as well as antibodies against the CGRP receptor and CGRP itself. The CGRP antagonists (BI 44,370 TA, MK-3207, olcegepant, imegepant, telcagepant, and ubrogepant) were shown to be effective as migraine abortive therapy [44,45,46,47,48,49,50]. Further, various antibodies against CGRP: eptinezumab, fremanezumab and galcanezumab proved to be effective for various forms of migraine, while retaining a safe therapeutic profile [51,52,53,54,55,56]. Similarly promising results were noted for erenumab (AMG 334), a monoclonal antibody against the CGRP receptor [57,58].

### 2.4. The Role of CGRP and Glial Cells in Migraine Pathogenesis

Since the mid-1980s, it has been postulated that CGRP integrally influences the neuron-glial interactions associated with migraine pain propagation [33]. CGRP is likely released from TG neurons in response to migraine triggers e.g., stress and hypoxia communicated via afferents from the periphery [59]. Moreover, although a direct link between CGRP and CSD has not been established, increased extracellular K^+^, seen as a condition for the latter, may drive CGRP release (Figure 2) [25]. CGRP triggers NO production which in turn leads to increased expression of CGRP and neuronal nitric oxide synthase (nNOS) [60] creating a positive feedback loop that promotes sensitization of primary peripheral trigeminal fibers and activity-independence of central second-order neurons [61]. These mediators further stimulate the surrounding glial cells, termed satellite cells, to produce interleukin-1β (IL-1β) which promotes increased cyclooxygenase activity, such that is tied to the production of proinflammatory prostaglandin E2 (PGE2) [62,63,64]. Similarly, CGRP-stimulated satellite cells release TNFα, which has also been implicated in the positive feedback driving TG neuron sensitization, both directly and via the release of additional proinflammatory cytokines [65,66]. Indeed, the literature suggests that the secretion of CGRP from one type of TG neuron may induce cytokine secretion in other TG neurons as well as in adjacent satellite glial cells [62,67,68]. It is the TG satellite cell-mediated up-regulation of specific proalgogenic receptors combined with long-term sensitization of TG neurons that enhances pain [61]. Further corroborating this hypothesis, a study by Cady et al. [69] demonstrated that CGRP injection into the rat temporomandibular joint led to formation of the activated phenotype of both microglia and astrocytes in the TNC. This phenomenon is thought to be responsible for maintaining the central sensitization of neurons involved in pain perception and is likely implicated in the progression from episodic to chronic migraine [61]. Conversely, Cornelison et al. [70] showed that administering CGRP into the cisternae of the rat brain lead to activation of astrocytes but not microglia, as the authors observed no change in the level of the microglial marker ionized calcium-binding adapter molecule (Iba1), but did note enhanced expression of the astrocytic markers glial fibrillary acidic protein (GFAP) and protein kinase A (PKA).

Several inhibitors of glial cell activation including naltrexone, naloxone, minocycline and ibudilast have been proposed as prophylactics against migraine [71]. However, clinical trial results published by Kwok et al. [66] demonstrated similar attack frequency and intensity, as well as no changes in allodynia, quality of life, medication use, or the secondary measures of headache in chronic migraine patients who received ibudilast for eight weeks. Still, comparable studies have not been performed in patients with episodic migraine, leaving the question of whether ibdulast could prove effective in preventing the transformation of episodic migraine into a chronic disorder unanswered.

The interactions between activated trigeminal neurons and adjacent glial cells which are mediated by gap junctions and paracrine signaling are likely also connected to the development of peripheral sensitization within the TG and other elements of migraine pathogenesis [64]. It is hypothesized that elevated expression and activity of gap junctions and pannexin (Panx) channels at the level of the sensory ganglia and TG neurons in inflammatory and neuropathic models of pain may lead to augmented excitation of sensory neurons. It is also worth noting, the gap junctions and Panx in glial cells may contribute to development of migraine with aura, as they facilitate the spreading of signals between satellite glial cells, including Ca^2+^ waves [72].

Ca^2+^ spreading among glia and the aberrant transport of other ions may be associated in particular with a certain subtype of migraine with aura, called familial hemiplegic migraine (FHM). Despite the fact that neuron-glia co-sensitization occurs often in the course of migraine, in FHM the role of CGRP seems limited, as shown by the studies of Hansen et al. [73]. This fuels the suspicion that disturbances in ion transport within the brain arise from CGRP-independent processes [74].

### 2.5. Familial Hemiplegic Migraine

As the TGVS richly expresses ion channels [30], the hypothesis that migraine headache may be a result of dysregulated nerve excitation due to one or more channelopathies is an attractive one. Moreover, migraine shares clinical similarities with other channelopathies, e.g., myotonia or periodic paralysis including frequency and duration of attacks, paroxysmal character, triggers for attack, and gender-related predilection for attack [74]. Genetic studies of FHM have also substantiated the hypothesis that migraine, or at least aurae arise as a result of ionopathy.

FHM, the most severe subtype of MA, is characterized by the presence of temporary unilateral hemiparesis (numbness and/or motor weakness). Usually the symptoms of FHM start in the first or second decade of life. They may be accompanied by cerebellar atrophy and disturbances in cerebral blood flow. Less frequent are atypical attacks with cerebellar signs, encephalopathy, coma, prolonged hemiplegia, epileptic seizure, confusion, or fever, with full recovery or nystagmus and ataxia between attacks [75].

FHM is a rare, genetically heterogeneous disease, inherited in an autosomal dominant pattern with approximately 70–90% penetrance [76]. Three causative genes for FHM have been identified: *CACNA1A* (FHM type 1), *ATP1A2* (FHM type 2) and *SCN1A* (FHM type 3) [77,78,79]. The *CACNA1A* gene encodes the α1A subunit of the P/Q-type high-voltage activated calcium channel. The *ATP1A2* gene encodes the catalytic α2 subunit of Na^+^/K^+^ ATPase, which is exclusively expressed in astrocytes where it maintains the electrochemical gradient of Na^+^ and K^+^ ions essential for transport of Ca^2+^ and glutamate. The Na^+^/K^+^ ATPase also regulates the reuptake of K^+^ and glutamate. The elevated level of K+ due to FHM2 mutations triggers CSD [80]. The *SCN1A* gene encodes the α subunit of the neuronal voltage-gated sodium channel (Na_v_1.1). Na_v_1.1 is mainly expressed in the cerebral cortex and spinal cord where it is responsible for the generation and propagation of action potentials. Mutations in FHM genes occur also in epileptic patients [81].

## 3. Structure and Functions of CaV2.1

Voltage-dependent Ca^2+^ channels are multiprotein complexes consisting of α1, α2δ, β and γ subunits [24]. The structural and functional diversity of VGCCs results from the multiple isoforms of each subunit, especially α1, different gating kinetics, and the many proteins with which they interact, often via transient, low-affinity molecular interactions (Table 1). CaV1, CaV2 and CaV3 are paralogs which have arisen through gene duplication events unaccompanied by speciation. CaV1 channels control synaptic integration and modulate NMDA receptor-mediated plasticity at the post-synapse, regulate enzyme activity and gene expression, and initiate excitation-contraction coupling. CaV2 channels mediate neurotransmission at the pre-synaptic active zone, while the CaV3 subfamily modulates the depolarization threshold for action potential initiation in cardiomyocytes and thalamic neurons. Both CaV1 and CaV2 are classified as high voltage-activated, while CaV3s are low voltage-activated channels [24,29,82].

Of all of the culprits potentially implicated in migraine channelopathy, the P/Q-type CaV2.1 channel has received the most attention. CaV2.1 channels are present in presynaptic terminals and somatodendritic membranes in the brain and spinal cord [83]. Importantly, they are expressed in brain regions responsible for nociception or even strongly implicated in migraine pathogenesis e.g., TG and brainstem and control the release of vasoactive neuropeptides in the TGVS [84].

**Figure 3:**
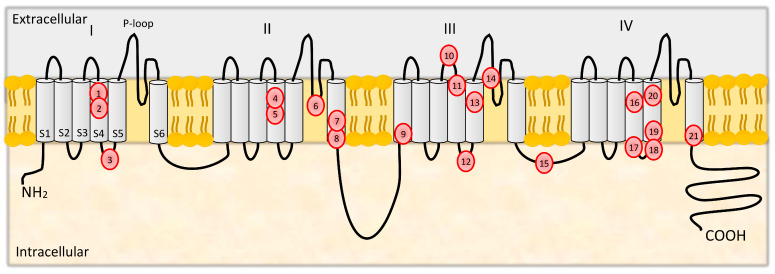
Locations of FHM1 mutations in the secondary structure of the calcium channel α2.1 subunit. 1—R192Q, 2—R195K, 3—S218L, 4—V581M, 5—R583Q, 6—T666M, 7—V714A, 8—D715E, 9—Y1246C, 10—K1336E, 11—R1347Q, 12—C1370Y, 13—Y1385C, 14—V1457L, 15—C1535S, 16—R1668W, 17—L1682P, 18—W1684R, 19—V1696I, 20—I1710T, 21—I1811L. I-IV- number of extracellular loop.

As presented in Figure 3, the α1A subunit of CaV2.1 is formed from four homologous domains (I–IV) consisting of six transmembrane regions (S1–S6). The S4 region constitutes the voltage sensor, while S5, the P-loop, and S6 form the pore region, which determines ion selectivity and conductance properties [24,29,82].

CaV2.1 channels control action-potential evoked neurotransmitter release, by triggering activation of the exocytotic machinery at the pre-synapse [25]. CaV2.1s are able to interact with numerous Ca^2+^-binding proteins [24] and maintain short-term plasticity via Ca^2+^-dependent inactivation and Ca^2+^-dependent facilitation. CaV2.1 channels are also involved in local excitability of neurons, Ca^2+^ signaling, cell survival or gene expression [85]. Remarkably, CaV2.1 channels facilitate both fast neurotransmission and modulation of neuromuscular transmission of acetylcholine mediated by muscarinic M1 and M2 receptors and protein kinases A and C [86,87].

### 3.1. Mutations in CACNA1A

The *CACNA1A* gene consists of 47 exons and is located on chromosome 19p13. Age and gender-dependent alternative splice variants of exon 37 were found, which correspond to differences in channel kinetics and confer subfunctionalities to isoforms expressed in different brain regions [88].

Mutations in *CACNA1A* are associated with a few neurological diseases, including FHM, episodic ataxia type 2 (EA2), spinocerebellar ataxia type 6 (SCA6) and nonprogressive congenital ataxia (NPCA), and epilepsy [89]. Patients with FHM may present with symptoms of ataxia. Mutations in FHM genes are not found in common forms of MA and MO. *CACNA1A* mutations are responsible for about half of FHM cases. 21 FHM1 mutations were identified, all of them missense mutations leading to substitutions of amino acids in functional regions of the CaV2.1 channel, with the majority occurring in transmembrane segments of the α_1_ subunit (Figure 3).

Generally, different mutations are associated with pure FHM1 and FHM1 with cerebellar symptoms. For example, the R192Q mutation is responsible for a mild form of FHM1, whereas the S218L mutation causes a severe, often lethal phenotype [90]. Moreover, individuals with the same mutation may differ in terms of clinical symptoms, which suggests that epigenetic and environmental factors may be involved in determining phenotype (Table 1) [75].

Mutations in *CACNA1A* may be divided into three groups: gain of function, loss of function, or biallelic mutations. Half of FHM1 cases are caused by gain of function mutations. 13 of FHM1 mutations (R192Q, S218L, R583Q, T666M, V714A, D715E, Y1246C, K1336E, V1457L, W1684R, V1696I, I1710T, I1811L) were investigated in heterologous expression systems expressing recombinant CaV2.1 channels [105,106,107,108,109,110,111,112,113,114,115,116,117]; some of them (R192Q, S218L, T666M, V714A, I1811L) were also studied in neurons from CaV2.1−/− mice expressing human CaV2.1 α1 subunits [108,111,118,119,120].

Studies on the HEK293 cell line [106] as well as on CaV2.1−/− neurons [108] evidenced that there is a decreased density of functional CaV2.1 in FHM1 mutant cells as compared to WT. This, however, has been rationalized as an artifact of overexpression post-transfection insofar as decreased numbers of functional channels were not observed when hCaV2.1 were expressed endogenously in knock-in mice [25]. Nonetheless, other studies on CaV2.1−/− neurons found no differences in synaptic strength [118,119]. Further, CaV2.1−/+ mice have a reduced response to neuroinflammatory and neuropathic pain due to having roughly half the number of CaV2.1 channels, albeit in an age-dependent manner. This is compatible with the idea that CaV2.1 channels may be pronociceptive in terms of inflammation and neuropathic pain and antinociceptive in response to acute non-injurious noxious thermal stimuli.

*CACNA1A* mutations result in a wide spectrum of consequences including increased channel open probability, a lower voltage of activation leading to enhanced Ca^2+^ influx, reduced channel inhibition by G-protein βγ heterodimers, altered synaptic morphology, excitation-inhibition imbalance and enhanced glutamate release. According to in vitro models of CSD, the increased glutamate release and interaction between glutamate and pre- and postsynaptic glutamate NMDA receptors may facilitate CSD. Importantly, *CACNA1A* mutations promoting increased Ca^2+^ influx seem to selectively capacitate glutamate release at pyramidal neurons, without altering fast-spiking inhibitory interneurons [117]. Further, the excitotoxic effects of glutamate on neuronal and glial cells have been shown to alter brain energy metabolism [117,120,121]. A study by Eikermann-Haerter et al. [115] showed that mice expressing the R192Q *CACNA1* mutation were more sensitive to CSD than those bearing the S218L variant. Moreover, female mutant mice were more susceptible to CSD and neurological deficits than males. The R192Q mouse model pointed not only to a decreased threshold for CSD but also increased CaV2.1 current density [122]. Susceptibility to CSD can also be heightened by female hormones and allele dosage. In addition, FHM1 mice showed greater oxygen consumption leading to tissue anoxia, which may be responsible for prolonged aura [117].

### 3.2. Calcium-Related Therapeutics in Migraine

Migraine pharmacotherapy includes prophylactic agents taken every day (e.g., antidepressants and antiepileptic drugs) and agents taken at headache onset (e.g., triptans). Nonetheless, migraine patients typically migrate to analgesics, typically nonsteroidal anti-inflammatory drugs (NSAIDs) available without prescription, despite mixed reports over their efficacy. Unfortunately, triptans, selective 5-HT1B/1D receptor agonists, which make up 80% of prescribed medications, proved to be effective in only 60% of migraine patients not responding to NSAIDs [123,124,125].

Unfortunately, most drugs currently in use do not prevent the recurrence of migraine attacks. Thus, migraine may be the cause of numerous absences at school, or at work. It was calculated that the costs of absenteeism from work due to migraines are much higher than the costs of its treatment. Important also is the fact that 3–4 times more funds are spent for the treatment of chronic migraine in comparison with the episodic subtype. It is estimated that in the USA the annual total costs incurred for migraine, taking into account both the treatment, as well as losses due to absence from work add up to as much as 13 billion USD [126]. The lack of efficacious analgesics or means to prevent migraine recurrence in many patients has created a need for finding novel therapeutics.

Currently, there are no selective small molecule inhibitors of CaV2.1. Only two peptide toxins are selective for CaV2.1: ω-agatoxin IVA and ω-agatoxin IVB isolated from the venom of American funnel web spider *A. aperta*. Both ω-agatoxins bind on the outside of the pore region of α1a subunit of CaV2.1 but have different kinetics: blocking by ω-agatoxin IVB is eightfold slower than IVA [127]. However, there are drugs that are not selective for but act also at CaV2.1 and show clinical efficacy in patients with FHM1 including flunarizine and the non-dihydropyridine verapamil [128].

New targets for migraine therapy may focused on different Ca^2+^ channels. One class worth noting is acid-sensing ion channels (ASICs), among which the ASIC1 predominates in the CNS. ASIC1 channels are responsible for enhancing Ca^2+^ permeability and pain signaling [129] and their overexpression has been found in chronic inflammatory and neuropathic models [130]. How ASICs are implicated in migraine pathogenesis remains largely unknown. Still, ASICs are activated by decreases in pH regulated in part by serotonin (5-HT) and nerve growth factor (NGF), changes in both of which are observed in migraine patients [131,132]. Secondly, ASIC1a is overexpressed in hypothalamic orexinergic neurons thought to be involved in migraine pathophysiology. Finally, lower pH secondary to ASIC activation may initiate or propagate CSD [129,132]. Corroborating this hypothesis, a study using experimental models showed that both amiloride (a nonselective blocker of ASICs) and tarantula toxin PcTx1 (a selective blocker of ASIC1a) inhibited CSD [133].

## 4. Conclusions

Migraine is a multifactorial neurological disease whose pathogenesis has not been fully elucidated. Although numerous dysregulated processes likely converge to manifest as migraine, disturbances in the distribution of various ions between extracellular and intracellular compartments seem to be a common denominator in the pathophysiology of the disease. Migraine ”ionopathy” likely derives from one or multiple channelopathies which may be inherited e.g., mutations in *CACNA1A* and *TRPA1*, or result from the effects of toxic environmental factors. Certain parts of the brain appear particularly impacted by migraine-associated channelopathies. The TG, which is the major afferent pathway for pain signals from cranial vessels and dura mater, is implicated in migraine pathogenesis largely owing to its release of neuropeptides such as CGRP. CGRP is one of the most important molecules involved in neuron-glial communication, whose breakdown is related to the propagation of migraine pain and the sensitization of neurons which likely precedes the progression from episodic to chronic migraine. How calcium channelopathies impact CGRP pathways however, remains largely unknown. CSD, a purportedly non-synaptic phenomenon, and gap-junctional interactions also play a role in MA by driving aberrant neuron-neuron and neuron-glia signal propagation. As is the case with other neurological disorders, the complex and still poorly understood pathogenesis of migraine makes treatment difficult. Nonetheless, the pivotal role of CGRP in the disease has made it a prominent therapeutic target.

## Figures and Tables

**Table 1:** Clinical presentation of *CACNA1* mutation in FHM.

Mutation	Location	Population	Phenotypic Spectrum	References
R192Q rs121908211	exon 4	Italian family	No data	Ophoff et al. [77]
R195K rs121908222	exon 4	French family	FHM without cerebellar signs	Ducros et al. [75]
S218L rs121908225	exon 5	British and Australian families, Malaysian family	Minor head trauma–triggered delayed severe cerebral edema and coma; childhood seizures	Kors et al. [90] Chan et al. [91]
V581M	exon 13	German family	FHM with cerebellar dysfunction and late-onset cognitive decline	Freilinger et al. [92]
R583Q rs121908217	exon 13	Italian family, Dutch patient, Portuguese family, Dutch patient	FHM with consciousness and fever lasting several days, late-onset cerebellar ataxia and cerebellar atrophy; symptoms triggered by minor head trauma; sporadic hemiplegic migraine without cerebellar signs, age at onset 13 years	Battistini et al. [93] Terwindt et al. [94] Alonso et al. [95] de Vries et al. [96]
T666M rs121908212	exon 16	American family, Australian family, French families, Dutch patient, Dutch families	Age at onset between 2 and 22 years; FHM with progressive cerebellar ataxia; sporadic hemiplegic migraine; progressive cognitive dysfunction	Ophoff et al. [77] Friend et al. [97] Ducros et al. [98] Terwindt et al. [94] Kors et al. [99]
V714A rs121908213	exon 17	British family	Age at onset between 10 and 21 years	Ophoff et al. [77]
D715E rs121908218	exon 17	French family	FHM with progressive cerebellar ataxia	Ducros et al. [98]
K1336E	exon 25	French family	FHM without cerebellar signs	Ducros et al. [75]
R1347Q rs121908230	exon 25	Dutch families	Wide clinical spectrum ranging from (trauma triggered) hemiplegic migraine with and without ataxia, loss of consciousness and epilepsy, early age at onset (usually before the age of 3)	Stam et al. [100]
Y1385C rs121908219	exon 26	French patients	FHM with cerebellar signs, coma, hyperthermia, meningeal signs, and partial seizure,	Vahedi et al. [101] Ducros et al. [75]
V1457L rs121908237	exon 27	Italian family	Mean age at onset 34 years, various degrees of aphasia congruent with the hemispheric dominance, without cerebellar ataxia or coma	Carrera et al. [102]
R1668W	exon 32	French family	FHM with or without cerebellar signs	Ducros et al. [75]
W1684R	exon 32	French family	FHM with cerebellar signs	Ducros et al. [75]
V1696I	exon 33	French family	FHM without cerebellar signs	Ducros et al. [75]
I1710T rs121909326	exon 33	Dutch family	FHM with childhood-onset of cerebellar ataxia (SCA6), childhood complex partial and generalized tonic-clonic seizures that occurred independently of the FHM attacks	Kors et al. [103]
I1811L rs121908214	exon 36	Dutch and American families	Only one family displayed cerebellar atrophy and in that family only some members were affected	Ophoff et al. [77]
18.2-kb deletion	exons 39–47	Irish, Indian, and Danish patients	FHM with or without ataxia, no seizures, no family history	Labrum et al. [104]

## Data Availability

Not applicable.
